# Effects of prebiotic (lactoferrin) and diclazuril on broiler chickens experimentally infected with *Eimeria tenella*

**DOI:** 10.3389/fvets.2024.1416459

**Published:** 2024-07-05

**Authors:** Asmaa G. Abd El Monsef, Nermin F. El Zohairy, Marwa F. Hassan, Sanaa M. Salem, Asmaa Aboelabbas Gouda, Mogda K. Mansour, Abdulsalam A. M. Alkhaldi, Hind Alzaylaee, Ehab Kotb Elmahallawy

**Affiliations:** ^1^Department of Biochemistry, Toxicology and Feed Deficiency, Animal Health Research Institute (AHRI), Agriculture Research Center (ARC), Zagazig Branch, Zagazig, Egypt; ^2^Department of Biochemistry, Toxicology and Feed Deficiency, Animal Health Research Institute (AHRI), Agriculture Research Center (ARC), Dokki, Giza, Egypt; ^3^Department of Pathology, Animal Health Research Institute (AHRI), Agriculture Research Center (ARC), Zagazig Branch, Zagazig, Egypt; ^4^Department of Parasitology, Faculty of Veterinary Medicine, Zagazig University, Zagazig, Egypt; ^5^Biology Department, College of Science, Jouf University, Sakaka, Saudi Arabia; ^6^Department of Biology, College of Science, Princess Nourah bint Abdulrahman University, Riyadh, Saudi Arabia; ^7^Departamento de Sanidad Animal, Grupo de Investigación en Sanidad Animal y Zoonosis (GISAZ), Universidad de Córdoba, Córdoba, Spain; ^8^Department of Zoonoses, Faculty of Veterinary Medicine, Sohag University, Sohag, Egypt

**Keywords:** anticoccidial, lactoferrin, diclazuril, *Eimeria tenella*, broiler chickens

## Abstract

**Introduction:**

Avian coccidiosis presents a significant challenge to the poultry industry in Egypt, highlighting the urgent need for validating new drug targets offering promising prospects for the development of advanced anticoccidials. Although numerous reports highlight the activity of lactoferrin (LF) against various microorganisms, its potential against *Eimeria* has not been explored. The present study evaluated the potential anticoccidial effect of LF and diclazuril in broiler chickens experimentally infected with *Eimeria tenella*.

**Methods:**

A total of 100 one-day-old broiler chicks were divided into five equal groups (20 each) as follows: Group 1 (G1) served as the normal healthy control group, Group 2 (G2) consisted of chickens infected with 1 × 10^5^ sporulated *E. tenella* oocysts at 14 days of age, Group 3 (G3) comprised infected chickens treated with diclazuril (0.5 mL/L in drinking water) for 3 days successively, Group 4 (G4) included infected chickens treated with LF (at a dose of 250 mg/kg of diet) from one day of age until the end of the study, and Group 5 (G5) comprised infected chickens treated with both LF and diclazuril.

**Results:**

The positive control group (G2) experienced significant reductions in body weight (BW), BW gain, serum glucose, lipase, amylase, total antioxidant capacity, several hematological indices, and total proteins, along with alterations in various antioxidant enzymes. Conversely, serum levels of aspartate aminotransferase (AST), Alanine aminotransferase (ALT), Alkaline phosphatases (ALP), urea, creatinine, nitric oxide, mean corpuscular volume (MCV), White blood cells (WBCs), heterophils, alpha 2, beta 1, and liver contents of malondialdehyde were elevated in this group. Moreover, higher oocyst counts and lesion scores, along with histopathological alterations, were observed in G2. Remarkably, treatment with diclazuril and/or LF demonstrated potent antioxidant and anticoccidial effects, resulting in reduced shedding of oocysts, lesion scores, and lymphocytic infiltrates in the cecum. Additionally, these treatments improved the antioxidant and immune systems in chickens and restored all histopathological changes reported in the infected non-treated group (G2).

**Conclusion:**

This study offers novel perspectives on the potential anticoccidial effects of the combination of LF and diclazuril in broiler chickens infected with *E. tenella*, highlighting the potential synergistic actions of LF in treating poultry coccidiosis.

## 1 Introduction

Avian coccidiosis remains one of the main diseases influencing poultry breeding, causing an annual economic loss of over 800 million US dollars worldwide (1). This disease is caused by intracellular protozoan parasites belonging to the genus *Eimeria* (2). Several species of *Eimeria* were identified in poultry, namely *Eimeria tenella, Eimeria mitis*, *Eimeria maxima*, *Eimeria acervulina*, *Eimeria brunetti*, *Eimeria necatrix,* and *Eimeria praecox* (3, 4). Their clinical effects on poultry range from subclinical infections to animal deaths. Among the others, *E. tenella*, and *E. necatrix* are classified as severe pathogens in poultry. Coccidiosis is most common and hard to manage, particularly in poultry breeding, leading to intestinal lesions and reduction of feed conversion, weight income, and egg production (5, 6). Considered individually, each *Eimeria* species may impact a specific region of the intestine; for instance, *E. tenella* primarily affects the cecum, while *E. acervulina* predominantly targets the duodenum (7). As aforementioned, the parasite is widely distributed in the environment and several strategies are used for its control, including anticoccidial agents. However, drug-resistant isolates of *Eimeria* spp. emerge using these drugs (1). Among others, diclazuril (benzeneacetonitrile) is a triazine-based antiprotozoal agent, containing 1,2,4-triazine, and has long been used to control *E. tenella* (8). This drug effectively hinders the sexual and asexual stages of coccidia by impeding the excretion of oocysts, which ultimately halts the lifecycle of these parasites (9). Moreover, diclazuril was proved to be a highly effective nucleotide analog anticoccidial agent resulting in a significant reduction of mean oocyst shedding and destructed all intracellular developmental stages of *E. tenella* (both schizonts and gamonts) (10). Diclazuril either alone or as compared with other anticoccidial agents in the ratio has the potential anticoccidial activity (11). Nevertheless, there has been a concerning rise in reports of resistance to diclazuril and other anticoccidial drugs. Additionally, the excessive use of these drugs may result in residues in animal products, posing potential public health hazards (8, 12). Therefore, searching for novel effective anticoccidial agents remains one of the main measures to control coccidiosis in poultry. Various previous reports explored the potential benefits of natural products and plants in the treatment of coccidiosis (13, 14).

Interestingly, prebiotics and probiotics are becoming increasingly important in preventing and controlling various infectious diseases including coccidiosis (15). A previous study (16) has noted that different dietary probiotic supplements can impact the host’s immunity against coccidiosis. Lactoferrin (LF) is an iron-binding glycoprotein that has the potential to enhance the pre-immune host defense system. It is derived from various sources, including secretory fluids such as maternal milk or tears, mucous secretions, and secondary granules of neutrophils and blood (17). LF has a unique structure and possesses potent antimicrobial and immunomodulatory activities related to the transferrin family (18, 19). Moreover, LF has previously been shown to improve the performance of poultry (20). The cellular and molecular mechanisms responsible for the immunomodulatory effects of LF are not fully elucidated. However, *in vitro,* and *in vivo* studies suggested the existence of multiple mechanisms, including modulation of cytokine/chemokine production, regulation of the production of reactive oxygen species, and immune cell recruitment (21). In addition to its antioxidant properties, LF is a natural iron scavenger, activator/modulator of signaling pathways, and targets the negative feedback of the inflammatory response (22–24). Moreover, the antimicrobial activity of LF seems related to iron deprivation through the removal of an essential substrate that is required for bacterial growth (25), while its bacteriostatic effect is mainly conducted by degrading the peptidoglycans in the bacterial cell wall affecting the membrane permeability resulting in cell lysis (26). While numerous studies have highlighted the protective properties of LF as an antibacterial and antifungal agent, limited research has investigated its potential activity against parasites, especially coccidia.

Upon reviewing the existing literature, it is evident that only a single previous study has explored the influence of bovine LF on *Eimeria stiedae in vivo* (mice and rabbits) and *in vitro* (mouse embryonic and rabbit hepatobiliary cells) (27). This study aimed to assess the potential anti-parasitic and immunomodulatory effects of dietary supplementation with LF, either alone or in combination with the widely used anticoccidial drug diclazuril, in broiler chickens infected with *Eimeria* spp. The methodology involved evaluating clinical signs, parasitological parameters, oxidative stress markers, hematological changes, alterations in immunological gene expression, and histopathological findings.

## 2 Materials and methods

### 2.1 Ethical consent

The experimental protocol was approved by the Animal Health Research Institute (AHRI) in conformity with the ARC and IACUC committee (ARC, AHRI, IACUC, 85/23).

### 2.2 Drugs

Diclazuril (Diclosol 1%), in a water-soluble formulation, was supplied by Pharma Sweed-Egypt company, located in 10th of Ramadan City, B3, No:2353/2004, and Code No. 205.87.20.v3. Prebiotic LF was obtained from Dulex Lab Nutrition and Pharmaceutical company, situated in New Cairo, Batch No: B2117051.

### 2.3 Parasite strains and parasite preparation

In this step, sporulated oocyst of *E. tenella* was obtained from the Parasitology unit, Poultry Diseases, and Research Department, Animal Health Research Institute (Dokki, Egypt). In this step, *E. tenella* oocysts were isolated from the caecum of naturally infected chicks following the protocols described elsewhere (28, 29). The isolated oocysts were allowed to sporulate at standard temperature in a 2.5% potassium dichromate solution. Subsequently, the concentrated sugar flotation method was employed to eliminate debris from the sporulated Eimeria oocysts, followed by three rinses with distilled water. The Mc-Master technique was then utilized to adjust the count to 1 × 10^5^ sporulated oocysts per 1 mL (28, 29).

### 2.4 Birds and experimental design

One hundred healthy one-day-old (unsexed) Cobb broiler chicks were procured from a hatchery located in El-Kahera Poultry Company, 10th of Ramadan City, Egypt. These chicks were provided with a balanced commercial diet obtained from Feed Mix company until they reached 7 days of age. Subsequently, they were transferred to wire-floored cages for the duration of the experiment, with strict adherence to hygienic standards. The chickens had unrestricted access to an antibiotic- and anticoccidial-free diet, along with *ad libitum* access to tap water. Optimal temperature conditions were maintained using electric radiators and ventilators. Fecal samples were examined twice daily during the first 14 days of the experiment to confirm the absence of *Eimeria* oocysts using the flotation technique with NaCl-saturated solution (specific gravity of 1.28). Additionally, all birds were vaccinated against Newcastle disease virus (NDV) using HitchnerB1 and LaSota vaccines at 7 and 18 days of age, respectively. They also received an infectious bursal disease vaccine at 15 days of age. The NDV vaccines were procured from Intervet Boxmeer Company in Holland, while the Gumboro vaccine was obtained from Rhone-Merieau Company in France. According to the experimental protocol (30, 31) outlined in Table 1, the chicks were randomly divided into five groups, each comprising 20 individuals, then they were subsequently divided into three biological replications, each containing 6–7 chickens (with 3 cages per group). Of these, 80 animals were infected, while the remaining 20 were kept uninfected. On the 14th day of age, chicks from all groups (G2-G5), excluding G1, were administered with 1 × 10^5^/mL sporulated *E. tenella* oocysts per chick in 1 mL PBS using an insulin syringe inserted directly into the crop of each bird.signs daily (10, 30, 32).

**Table 1:** The experimental protocol and groups.

Group order	Treatment strategy
Group 1 (G1)	Negative control group (non-infected and non-treated/healthy group).
Group 2 (G2)	Positive control group infected with *Eimeria tenella* at age of 14th days (infected non-treated group) (10, 32).
Group 3 (G3)	Infected with *Eimeria tenella* at age of 14th days and treated with 5 ppm diclazuril in the drinking water (0.5 mL/L) at age of 19^th^ days for 3 successive days in drinking water (30).
Group 4 (G4)	Chicks treated with lactoferrin (250 mg/kg of diet) from 1st day of age until the end of experimental protocol and infected with *Eimeria tenella* at age of 14th days (31).
Group 5 (G5)	Chicks received lactoferrin (250 mg/kg of diet) from 1st day of age until the end of experimental protocol and infected with *Eimeria tenella* at age of 14th then treated with diclazuril (5 ppm) in the drinking water (0.5 mL/L) at age of 19^th^ days for 3 successive days in drinking water (30, 31).

### 2.5 Assessment of clinical signs and mortality rate

Clinical signs and the number of dead chicks in the infected and treated groups were recorded daily throughout the experimental period. The Mortality rate is calculated as a percentage according to the formula.


$$\begin{array}{l} \mathrm{Mortality rate}\;\left(\%\right)=(\mathrm{number of dead chicks in}\;a\;\mathrm{specified}\\ \;\;\;\;\;\;\;\;\;\;\;\;\;\;\;\;\;\;\;\;\;\;\;\;\;\;\;\;\;\;\;\;\;\;\;\;\;\;\;\;\;\mathrm{period}\times 100)/\mathrm{total number of}\;\mathrm{all}\;\mathrm{chicks} \end{array}$$

### 2.6 Assessment of growth performance

This step involved assessing the weekly body weight (BW) and body weight gain (BWG), which were recorded throughout the 22-day study period.

### 2.7 Sampling

**Figure 1:**
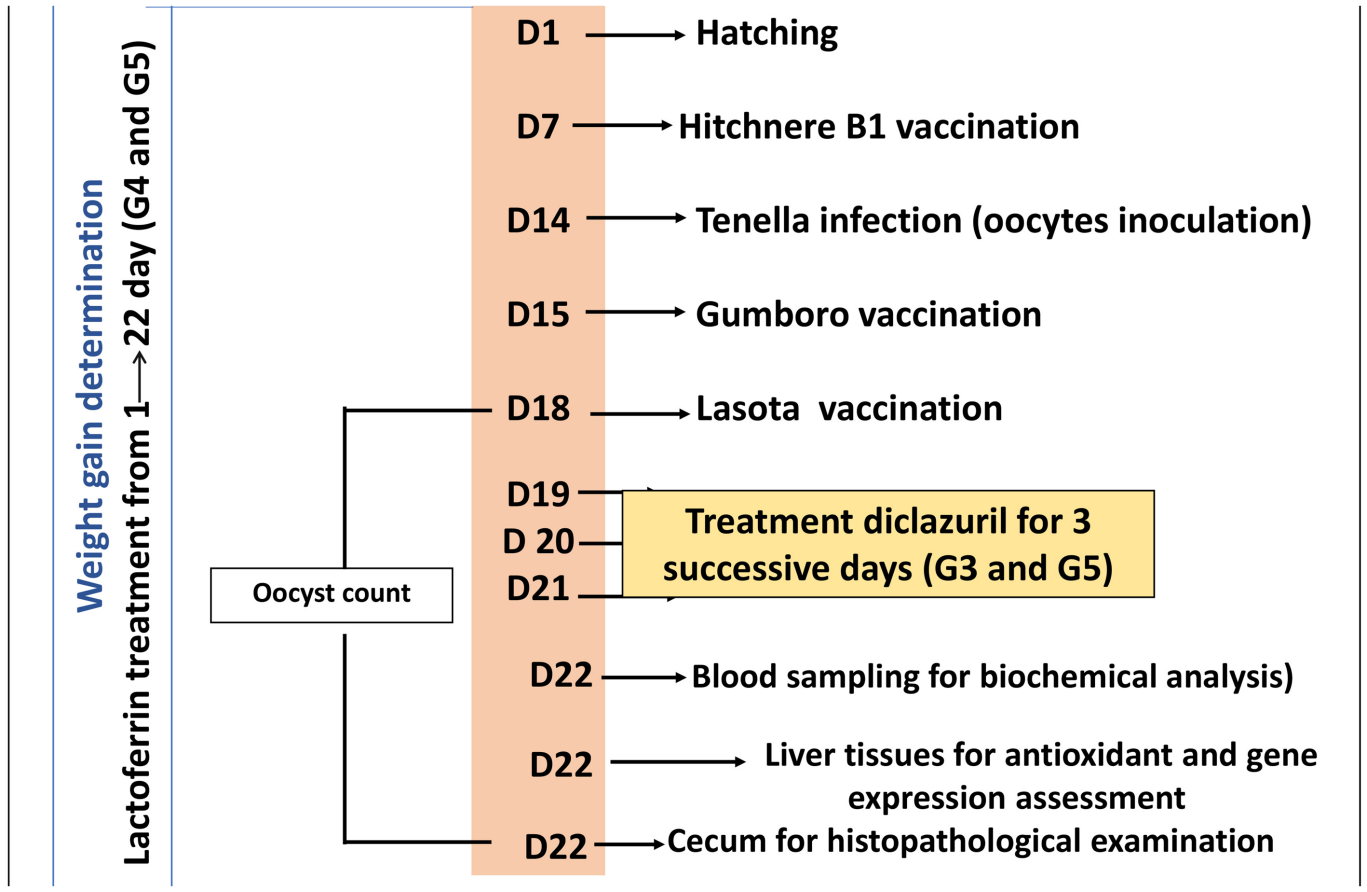
A timeline detailing the experimental protocol, including the day (D) number, treatments, vaccination, and sampling procedures.

On day 22 of the experiment, chicks were then sacrificed by neck dislocation and blood, liver, and cecum tissue samples were collected. Five blood samples were collected by wing vein from each group. Each blood sample was divided into two separate tubes. The first tube contained EDTA as an anticoagulant for complete blood count analysis, while the second tube did not contain any anticoagulant and was used for obtaining serum. Serum samples were obtained by centrifuging the blood samples at 3000 rpm for 5 min. Sera were isolated and stored at −20°C for biochemical analysis. Regarding tissue samples, liver tissue was excised and divided into two sections: one for antioxidant assessment and the other for gene expression analysis. Additionally, caecum tissue was excised and prepared exclusively for histopathological examination. The experimental treatments and sampling timeline are elucidated in Figure 1.

### 2.8 Evaluation of anticoccidial efficacy of prebiotic (lactoferrin) and diclazuril

This step involved the evaluation of the fecal oocyst output. Fresh fecal sample from each experimental group was daily collected in a clean plastic bag for parasitological examination. *Eimeria* oocyst counting started from the fourth -day post-infection till the end of the experiment. Oocysts were counted in 1 g of feces using the McMaster-chamber method (14, 33).

### 2.9 Histopathological examination

Collected specimens from cecum were fixed in 10% neutral buffered formalin for 24 h, dehydrated in graded ethanol, cleared in xylene, embedded in paraffin, and sectioned at 5 μm thick tissue sections, then stained with hematoxylin and eosin (H&E) and examined microscopically for any histopathological alterations (34). All section photographs were taken using a Swift microscope associated with a Swift digital camera. The histopathological lesions were scored and estimated by semiquantitative methods as follows: “- = absence of lesion, + = 5–25%, ++ = 26–50%, and +++ = > 50%” (35).

### 2.10 Biochemical analysis

Manual quantification of erythrocyte (RBCs 10^6^/mm^3^) and white blood cell count (WBCs) 10^3^/mm^3^ were evaluated according to the method proposed by Feldman et al. (36). Hemoglobin (Hb) concentration (g/dL) and the percentage of packed cell volume (PCV %) were also estimated (37, 38). Two blood smears were prepared promptly after collecting each blood sample. Stained blood films using Giemsa stain were used for differential leucocyte count according to Anderson and Latimer (39). Alanine aminotransferase (ALT) and aspartate aminotransferase (AST) activities were estimated according to Reitman and Frankel (40). Additionally, alkaline phosphatase was assayed by the method described by El-Aaser and El-Merzabani (41). Urea level was measured following Wybenga et al. (42), creatinine level according to Henry (43), and glucose level was determined according to Tietz (44). Moreover, serum levels of lipase and amylase were estimated according to Lopez (45), while nitric oxide (NO) and total antioxidant capacity (TAC) levels according to Montgomery and Dymock and Koracevic et al. (46, 47), respectively. Liver content of malonaldehyde (MDA), superoxide dismutase (SOD) and catalase (CAT) activities, and reduced glutathione (GSH) levels were estimated according to Okhawa et al. (48), Nishikimi et al. (49), Aebi (50), and Beutler et al. (51), respectively. Meanwhile, serum total protein and their electrophoretic pattern were estimated according to Sonnenwirth et al. (52) and Davis (53), respectively, then calculated according to SynGene S. No. 17292^*^14518 sme^*^mpcs.

### 2.11 Antioxidative parameter analysis

Liver levels of malondialdehyde (MDA), superoxide dismutase (SOD), catalase (CAT) activities, and glutathione (GSH) levels were assessed using a colorimetric method with the Bio-diagnostic kit (Catalog numbers: MD 25 29, SD 25 21, CA 25 17, and GR 25 11, respectively). The protocols for these measurements were followed as described in the literature (48–51). Liver tissues were excised, weighed, and cut into small pieces, homogenized with a glass homogenizer into 9 volumes of ice-cold 0.05 mM potassium phosphate buffer (pH 7.4) to prepare 10% homogenates. The homogenized tissues were then centrifuged at 3000 rpm/15 min at 4°C then the resultant supernatant was used for the determination of the following parameters: SOD, CAT, and MDA. Approximately 0.1 g of liver tissues were minced into small pieces homogenized with a glass homogenizer in 0.2 mL of 25% metaphosphoric acid (MPA) (ref. No.: 253-433-4, Sigma-Aldrich, Germany), then 0.7 mL of distilled water was added, mixed, and incubated for 1 h and centrifuged for 10 min at 3,000 rpm then the clean supernatant was removed and used for determination of GSH concentration.

### 2.12 Transcription of target genes (IL-2 and IFN- γ) using qRT-PCR

This step involved the preparation of liver tissues from experimental groups which were collected, placed in the Eppendorf tubes, immediately kept in liquid nitrogen, and stored at −80°C till RNA extraction for determination of the gene expression of interleukin-2 (IL-2) and interferon-gamma (IFN-γ). The mRNA expression of IL-2 and IFN-γ in liver tissue was then estimated via quantitative real-time PCR (qRT-PCR) that was performed in the Biotechnology Unit, Animal Health Research Institute, Zagazig branch (Egypt). Extraction of RNA from liver tissue samples was done using the QIAamp RNeasy Mini kit (Qiagen, Germany, GmbH). Primers used were obtained from Metabion (Germany) and are shown in Table 2. Analysis of the SYBR green rt-PCR results was determined through the amplification curves and threshold cycles (Ct) values, which were estimated using the step one software. To evaluate the change of gene expression on the RNA of the samples, the CT of each sample was compared with that of the control group depending on the “ΔΔCt” method explained by Yuan (56) depending on the following ratio: (2^-ΔΔct^).

**Table 2:** Primers sequences of target genes.

Target gene	Primers sequences 5′- 3′	Length/bp	Reference
B- actin	CACCACAGCCGAGAGAGAAAT	135	(54)
	TGACCATCAGGGAGTTCATAGC		
IFN-γ	GT AAG GAA CTT CAG CCA TTG	670	(55)
	GAC GAA TGA ACT TCA TCT GCC		
IL-2	TGC TTT TAA CCG TCT TTG	659	
	GAT GCT CCA TAA GCT GTA		

### 2.13 Statistical analysis

The data were statistically analyzed by the one-way Analysis of Variance (ANOVA) test. Results were given as mean ± standard error using SPSS 14.0 (2006) followed by Duncan’s test. Statistical significance was set at *p* < 0.05.

## 3 Results

### 3.1 Direct anticoccidial effect of LF

#### 3.1.1 Clinical signs, lesion scoring, and mortality rate

In the present work, chickens of G1 served as healthy normal control negative group (non-infected) and therefore showed no signs during the experimental period. Meanwhile, chickens infected with *E. tenella* and non-treated (G2; control positive group) showed loss of appetite, dullness, depression, weight loss, ruffled feathers, dehydration, and bloody diarrhea that appeared on the fifth day after infection. Regarding the recorded mortality rate, the infected non-treated group (G2) exhibited a notable mortality rate of 30%, whereas none of the other groups reported any fatalities. In contrast, infected chickens treated with LF or diclazuril (G3-G5) displayed less serious signs and clinical signs gradually improved from the fifth day after infection and showed no mortalities in all these treated groups.

#### 3.1.2 Assessment of growth performance

The obtained results for the assessment of growth performance are demonstrated in Table 3, revealing a significant (*p* < 0.001) decrease in BW and BWG (14 to 22 days or 0 to 22 days) in infected non-treated chickens (G2) compared with normal control chickens (G1). In contrast, chickens that received diclazuril and/or LF (G3, G4, and G5) displayed a significant (*p* < 0.001) increase in BW and BWG when compared with the infected non-treated chickens (G2). It is notable that the LF supplementation led to increase in BW and BWG across the entire duration of the study, as observed in both the G4 and G5 groups.

**Table 3:** Effect of diclazuril and/or lactoferrin on body weight and body weight gain (in gram) during *E. tenella* infected of broiler chickens.

Groups Parameters	G1: Control healthy group	G2: Infected non-treated	G3: Infected + Diclazuril	G4: Infected + LF	G5: Infected + LF + Diclazuril	*p*
Initial body weight (0 days)	39.33 ± 0.33^a^	39.67 ± 0.67^a^	39.00 ± 0.58^a^	39.00 ± 0.58^a^	39.67 ± 0.88^a^	0.886
Body weight week 1 (7 days)	147.33 ± 2.40^b^	151.33 ± 2.40^b^	153.33 ± 4.06^b^	172 ± 3.06^a^	173.33 ± 5.7^a^	<0.01^**^
Body weight week 2 (14 days)	466.67 ± 9.82^b^	466.67 ± 5.46^b^	475.33 ± 1.45^b^	514 ± 5.86^a^	520.33 ± 3.76^a^	<0.001^***^
Final body weight week 3 (22 days)	1002.67 ± 2.90^c^	836.67 ± 7.86^d^	1010.33 ± 15.84^c^	1050.67 ± 6.36^b^	1128.67 ± 7.54^a^	<0.001^***^
Body weight gain from 14 to 22 days	536 ± 9.17^b^	370 ± 6.93^c^	535 ± 14.50^b^	536.67 ± 5.49^b^	608.33 ± 11.29^a^	<0.001^***^
Body weight gain from 0 to 22 days	963.33 ± 2.60^c^	797.00 ± 8.33^d^	971.33 ± 16.33^c^	1011.67 ± 6.69^b^	1089.00 ± 7.37^a^	<0.001^***^

Data are presented as (Mean ± SE). *n* = 3 chickens; Mean values with different superscript letters in the same raw are significantly different at (*p* ≤ 0.05). *Statistically significant at *p* ≤ 0.05, **Statistically significant at *p* ≤ 0.01, ***Statistically significant at *p* ≤ 0.001.

#### 3.1.3 Count of oocysts

The results in Table 4 showed that the first *E. tenella* oocysts appeared in faces on the 4th day post-infection, reached their peak at the 4-5th day post-infection (108 h post infection) and then declined again gradually until the 8th day post infection. The using diclazuril only for 3 successive days just following the appearance of blood in the dropping (fifth-day post-infection) in experimentally infected broilers showed a significant decrease in the number of oocysts shedding when compared with *E. tenella* non-treated group. In addition, using diclazuril and/or LF had an effective role in decreasing the number of oocysts. A highly significant reduction in the number of oocyst shedding was recorded using a combination of LF and diclazuril (Table 4).

**Table 4:** The curative efficacy of prebiotic (lactoferrin) and diclazuril on oocyst count (x10^3^ gm feces) from 5th to 9th day post experimentally infection in broiler chickens infected by *E. tenella*.

Time	Age in days	G2: Infected non-treated	G3: Infected + Diclazuril	G4: Infected + LF	G5: Infected + LF + Diclazuril	*F*	*p*
4th DPI	18th day	448.4^a^ ± 8.35	433.7^a^ ± 3.84	314.7^b^ ± 2.91	299.7^b^ ± 2.60	242.55^*^	<0.001^*^
5th DPI	19th day	163.7^a^ ± 3.84	146.7^b^ ± 4.48	133.0^bc^ ± 1.53	127.7^c^ ± 3.76	20.141^*^	<0.001^*^
6th DPI	20th day	134.7^a^ ± 1.76	64.33^c^ ± 2.91	98.67^b^ ± 3.53	45.0^d^ ± 2.31	212.68^*^	<0.001^*^
7th DPI	21st day	83.33^a^ ± 1.76	43.67^b^ ± 1.45	77.33^a^ ± 3.48	31.67^c^ ± 1.20	135.65^*^	<0.001^*^
8th DPI	22nd day	41.0^a^ ± 1.15	22.33^bc^ ± 1.45	33.67^ab^ ± 1.45	21.67^c^ ± 4.70	12.637	0.002^*^

Data were expressed using (Mean ± SE), F: F for One way ANOVA test, Pairwise comparison bet. Each 2 groups was done using Post Hoc Test (Tukey), *p*: *p* value for comparing between the studied groups, Means within the same raw having different letters ^a–d^are significant, *Statistically significant at *p* ≤ 0.05; DPI, Days Post infection.

#### 3.1.4 Histopathology

**Figure 2:**
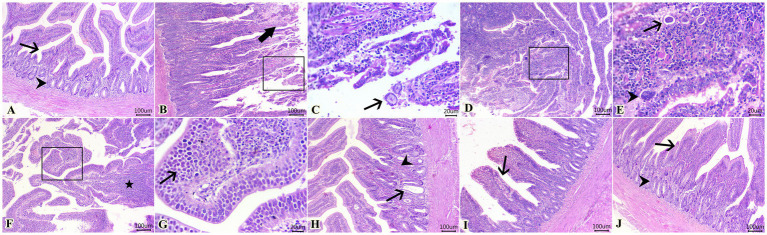
Photomicrographs of H&E-stained sections of cecum showing: **(A)** Normal histological architecture of cecal mucosa (arrow), crypts (arrowhead), submucosa, and muscular layer in G1. **(B,C)** Presence of necrotic enterocytes, desquamated sheets of epithelium (thick arrow), and macrogametes within destructed enterocytes (arrow). **(D,E)** Macrogametes (arrow) and microgametes (arrowhead) within the lamina propria. **(F,G)** Heavy infestation of some epithelial lining mucosa with large numbers of macrogametes (arrow) accompanied by lymphocytic infiltrates within the lamina propria (star) in G2. **(H)** Cystic dilatation of some cecal crypts (arrow), mild hemorrhage adjacent to eosinophils aggregation (arrowhead), and diffuse mononuclear cell infiltration within the lamina propria in G3. **(I)** Goblet cell hyperplasia (arrow), diffuse mononuclear cell infiltration, and mild hemorrhages in the lamina propria in G4. **(J)** Restoration of normal histological structures of cecal epithelium (arrow) and crypts (arrowhead) with mildly infiltrated lamina propria and submucosa with round cells in G5. Scale bar 100 μm. **(C,E,G)** Scale bar 20 μm.

The scoring of lesions based on the severity extent in cecal tissues among different experimental groups is depicted in Table 5. As illustrated, experimental infection by *E. tenella* in the control positive group (G2) highly induced severe lesions that received the highest score as compared with the normal healthy group (G1). In contrast, as shown in Table 5, diclazuril and LF significantly caused improvement in the histopathological lesion when compared with the infected non-treated group (G2). Regarding the histopathological findings, G1 showed normal histological architectures of cecal mucosa, crypts, submucosa, and muscular layer (Figure 2A). In contrast, G2 (Figures 2B–G) showed necrotic typhlitis represented by necrotic enterocytes, desquamated sheets of epithelium, intense lymphocytic infiltrates within lamia propria and submucosal layers besides extravasated erythrocytes. The presence of various developmental stages of coccidia within enterocytes and lamina propria (macrogametes, microgametes, and oocysts) was also encountered (Figures 2B,C). In this group, macrogametes were characterized by peripheral eosinophilic granules and the microgamete contained numerous basophilic nuclei (Figures 2D,E). Likewise, some epithelial lining mucosae were heavily infested by large numbers of macrogametes with intense infiltrations of lamina propria with round cells (Figures 2F,G). In contrast, the infected group treated with diclazuril (G3) showed cystic dilatation of some cecal crypts beside mild hemorrhage, eosinophils aggregation, and diffuse mononuclear cells infiltration within lamina propria (Figure 2H). Moreover, infected animals treated with LF treated group (G4) exhibited normal most cecal architectures with goblet cells hyperplasia, diffuse mononuclear cell infiltration, and mild hemorrhages at lamina propria (Figure 2I). Interestingly, the last group (G5) treated with a combination of diclazuril and LF G5 showed restoration of the normal histological structures of cecal epithelium and crypts with very mild infiltration of lamina propria and submucosa with round cells (Figure 2J).

**Table 5:** Scoring of severity and extent of lesions in cecal tissues among experimental groups.

Lesions	G1: Control healthy group	G2: Infected non-treated	G3: Infected + Diclazuril	G4: Infected + LF	G5: Infected + LF + Diclazuril
Necrotic typhlitis	−	+++	+	+	−
Goblet cells hyperplasia	−	+++	+	++	+
Developmental stages of Coccidia	−	+++	+	+	−
Cystic dilatation of cecal crypts	−	++	++	−	−
Hemorrhages	−	++	+	+	−
Eosinophil aggregations	−	+++	++	+	+
Diffuse round cells infiltration	−	++	+	++	+

Number of examined fields (five fields/chicken, X100). The lesions were graded by estimating the percentage area affected in the entire section: Lesions score system was as follows: − = absence of lesion, + = 5–25%, ++ = 26–50%, and +++ = ˃50%.

### 3.2 Indirect protective effect of LF

#### 3.2.1 Assessment of blood profile and biochemical and antioxidant parameters in the serum

The recorded data in Table 6 illustrated that a significant (*p* < 0.001) increase in MCV, WBCs, and heterophil% was associated with a non-significant increase in basophil% in G2 as compared with G1. In stark contrast, all treated groups (G3-G5) showed a significant decrease in all previous parameters when compared with infected non-treated chickens (G2). In contrast, a significant (*p* < 0.05) decrease in RBCs, Hb, PCV, PLT, MCH, MCHC, lymphocyte%, and monocyte% was noted beside a non-significant decrease in eosinophil% in the *E. tenella* non-treated group (G2) when compared with the control healthy group. All treated groups (G3-G5) displayed a significant increase in all previous parameters as compared to G2 with infected non-treated chickens (G2).

**Table 6:** Effect of diclazuril and/or lactoferrin on blood profile during *E. tenella* infected of broiler chickens.

Groups Parameters	G1: Control healthy group	G2: Infected non-treated	G3: Infected + Diclazuril	G4: Infected + LF	G5: Infected + LF + Diclazuril	*p*
RBCsx10^6^/cmm	3.37 ± 0.19b^c^	2.70 ± 0.05^d^	3.49 ± 0.07^b^	3.09 ± 0.03^c^	5.04 ± 0.13^a^	<0.001^***^
Hb (g/dl)	12.42 ± 0.09^a^	8.82 ± 0.05^d^	11.50 ± 0.50^b^	9.89 ± 0.01^c^	13.11 ± 0.02^a^	<0.001^***^
Pcv %	31.71 ± 0.08^b^	29.18 ± 0.02^c^	31.34 ± 0.19^b^	29.14 ± 0.08^c^	32.79 ± 0.32^a^	<0.001^***^
PLT X10^3/cmm	265.33 ± 10.35^bc^	192.00 ± 9.24^d^	283.00 ± 8.08^b^	247.67 ± 8.37^c^	325.33 ± 8.95^a^	<0.001^***^
MCV (fl)	94.64 ± 5.65^b^	108.14 ± 2.06^a^	89.93 ± 1.33^b^	94.31 ± 1.15^b^	65.22 ± 2.32^c^	<0.001^***^
MCH (pg)	37.03 ± 1.90^a^	32.70 ± 0.79^b^	32.95 ± 0.76^b^	32.01 ± 0.28^b^	26.05 ± 0.63^c^	<0.001^***^
MCHC (g/dl)	39.16 ± 0.31^a^	30.23 ± 0.18^d^	36.68 ± 1.38^b^	33.94 ± 0.12^c^	39.98 ± 0.45^a^	<0.001^***^
WBCs x103/cmm	22.24 ± 0.10^b^	23.79 ± 0.36^a^	21.66 ± 0.24^bc^	21.13 ± 0.06^c^	20.20 ± 0.05^d^	<0.001^***^
Lymphocyte %	10.86 ± 0.12^a^	7.05 ± 0.18^b^	7.03 ± 0.09^b^	6.64 ± 0.03^b^	5.06 ± 0.17^c^	<0.001^***^
Heterophil %	9.92 ± .05^d^	15.50 ± 0.15^a^	13.31 ± 0.25^c^	13.06 ± 0.01^c^	14.03 ± 0.26^b^	<0.001^***^
Monocyte %	1.07 ± 0.04^a^	0.89 ± 0.03^b^	1.02 ± 0.07^ab^	1.07 ± 0.03^a^	0.89 ± 0.03^b^	<0.05^*^
Eosinophil %	0.31 ± 0.03^a^	0.27 ± 0.01^ab^	0.22 ± 0.03^bc^	0.28 ± 0.00^ab^	0.16 ± 0.01^c^	<0.01^**^
Basophil %	0.07 ± 0.00^ab^	0.08 ± 0.01^ab^	0.07 ± 0.00^ab^	0.09 ± 0.01^a^	0.06 ± 0.00^b^	0.102

Data are presented as (Mean ± SE). *n* = 3 chickens; Mean values with different superscript letters in the same raw are significantly different at (*p* ≤ 0.05). *p ≤ 0.05, **p ≤ 0.01, ***p ≤ 0.001.

As depicted in Table 7, the infected non-treated group (G2) experienced a significant (*p* < 0.05) decrease in serum total protein; albumin, alpha 1, total alpha, beta 2, total beta, gamma 1, gamma 2, total gamma, and total globulin and a non-significant decrease in alpha 2 and beta 1 compared with the normal group (G1). Meanwhile, treatment of infected chickens with diclazuril and/or LF (G3, G4, and G5) showed a significant (*p* < 0.05) increase in all these parameters as compared with G2. Associated with a non-significant increase in the A:G ratio in all experimental groups when compared with the normal group.

**Table 7:** Effect of diclazuril and/or lactoferrin on T. protein and its electrophoresis fractions and subfractions (g/dl) in broiler chickens experimentally infected with *E. tenella.*

Groups Parameters	G1: Control healthy group	G2: Infected non-treated	G3: Infected + Diclazuril	G4: Infected + LF	G5: Infected + LF + Diclazuril	*p*
T. protein	4.39 ± 0.15^a^	3.05 ± 0.09^c^	3.83 ± 0.17^b^	3.47 ± 0.22^bc^	3.67 ± 0.21^b^	<0.01^**^
Albumin	1.05 ± 0.03^a^	0.77 ± 0.03^c^	0.99 ± 0.07^ab^	0.84 ± 0.06^bc^	0.90 ± 0.05^abc^	<0.05^*^
Alpha 1	0.93 ± 0.04^a^	0.63 ± 0.01^b^	0.72 ± 0.01^ab^	0.70 ± 0.10^ab^	0.86 ± 0.16^ab^	0.167
Alpha 2	0.40 ± 0.03^a^	0.37 ± 0.04^a^	0.48 ± 0.03^a^	0.38 ± 0.10^a^	0.37 ± 0.08^a^	0.713
Total alpha	1.34 ± 0.03^a^	0.99 ± 0.03^c^	1.19 ± 0.02^abc^	1.08 ± 0.10^bc^	1.23 ± 0.08^ab^	<0.05^*^
Beta 1	0.40 ± 0.02^a^	0.34 ± 0.01^a^	0.39 ± 0.03^a^	0.38 ± 0.03^a^	0.37 ± 0.03^a^	0.508
Beta 2	0.34 ± 0.01^a^	0.25 ± 0.02^b^	0.35 ± 0.01^a^	0.31 ± 0.02^a^	0.35 ± 0.01^a^	<0.01^**^
Total beta	0.74 ± 0.02^a^	0.59 ± 0.02^b^	0.75 ± 0.03^a^	0.70 ± 0.04^a^	0.71 ± 0.03^a^	<0.05^*^
Gamma 1	0.99 ± 0.11^a^	0.51 ± 0.02^b^	0.63 ± 0.03^b^	0.62 ± 0.02^b^	0.60 ± 0.04^b^	<0.01^**^
Gamma 2	0.27 ± 0.03^a^	0.19 ± 0.01^b^	0.26 ± 0.01^a^	0.22 ± 0.03^ab^	0.24 ± 0.00^ab^	0.075
Total gamma	1.26 ± 0.14^a^	0.69 ± 0.03^b^	0.89 ± 0.05^b^	0.84 ± 0.04^b^	0.83 ± 0.04^b^	<0.01^**^
Total globulin	3.34 ± 0.15^a^	2.28 ± 0.07^c^	2.84 ± 0.09^b^	2.62 ± 0.16^bc^	2.77 ± 0.16^b^	<0.01^**^
A:G ratio	0.32 ± 0.02^a^	0.34 ± 0.01^a^	0.35 ± 0.02^a^	0.32 ± 0.01^a^	0.33 ± 0.01^a^	0.433

Data are presented as (Mean ± SE). *n* = 3 chickens; Mean values with different superscript letters in the same raw are significantly different at (*p* ≤ 0.05). *p ≤ 0.05, **p ≤ 0.01, ***p ≤ 0.001.

Furthermore, our data established in Table 8 revealed that serum AST, ALT, ALP, urea, creatinine, NO, and levels of MDA in the liver were significantly (*p* < 0.001) increased in *E. tenella* non-treated chickens when compared with the normal group. Meanwhile, all treated groups showed a significant (*p* < 0.001) decrease in all previous parameters when compared with *E. tenella* non-treated chickens. In contrast, a significant (*p* < 0.01) decrease in serum glucose; lipase (*p* < 0.01); amylase; TAC; and liver tissue SOD, CAT, and GSH levels were noted in the *E. tenella* non-treated group when compared with the normal control group. All treated groups displayed a significant increase in all previous parameters compared with the *E. tenella* non-treated chickens with the best results in G5.

**Table 8:** Effect of diclazuril and/or lactoferrin on biochemical and oxidative stress markers during *E. tenella* infected broiler chickens.

Groups Parameters	G1: Control healthy group	G2: Infected non-treated	G3: Infected + Diclazuril	G4: Infected + LF	G5: Infected + LF + Diclazuril	*p*
ALP (IU/L)	153 ± 6.35^d^	367.67 ± 6.39^a^	248.67 ± 7.26^b^	266.33 ± 10.98^b^	210.33 ± 4.06^c^	<0.001^***^
AST (U/L)	35.33 ± 2.19^e^	140 ± 2.65^a^	99.66 ± 4.41^b^	79.33 ± 6.64^c^	60 ± 4.04^d^	<0.001^***^
ALT (U/L)	22 ± 2.08^c^	57.33 ± 3.76^a^	35.33 ± 0.88^b^	40.33 ± 1.86^b^	36.67 ± 2.03^b^	<0.001^***^
Creatinine (mg/dl)	0.38 ± 0.04^d^	0.96 ± 0.02^a^	0.73 ± 0.03^bc^	0.77 ± 0.04^b^	0.65 ± 0.02^c^	<0.001^***^
Urea (mg/dl)	7.37 ± 0.47^c^	28.40 ± 0.35^a^	16.73 ± 1.19^b^	15.80 ± 1.01^b^	13.90 ± 1.31^b^	<0.001^***^
Glucose (mg/dl)	211.82 ± 9.31^a^	143.99 ± 4.13^c^	148.46 ± 8.81^c^	181.33 ± 3.76^b^	198.53 ± 1.59^ab^	<0.001^***^
Lipase (U/L)	32.98 ± 1.83^cd^	25.13 ± 0.59^d^	39.50 ± 1.78^bc^	46.68 ± 1.18a^b^	55.41 ± 8.29^a^	<0.01^**^
Amylase (U/L)	330.38 ± 6.73^a^	226.89 ± 6.50^b^	319.89 ± 0.44^a^	323.61 ± 0.36^a^	324.42 ± 3.12^a^	<0.001^***^
NO (μmol/L)	15.17 ± 0.48^d^	38.39 ± 0.60^a^	27.81 ± 1.33^b^	28.29 ± 0.38^b^	20.15 ± 0.98^c^	<0.001^***^
TAC (Mm/l)	2.15 ± 0.08^a^	1.38 ± 0.04^d^	1.65 ± 0.09^c^	1.90 ± 0.07^b^	1.94 ± .032^ab^	<0.001^***^
MDA (nmol/g)	3.67 ± 0.09^c^	6.99 ± 0.47^a^	5.40 ± 0.30^b^	5.22 ± 0.13^b^	4.52 ± 0.61^bc^	<0.01^**^
SOD (U/g tissue)	105.67 ± 3.38^a^	66.61 ± 0.99^e^	83.55 ± 1.68^c^	93.40 ± 2.05^b^	74.67 ± 1.35^d^	<0.001^***^
CAT (U/g)	8.96 ± 0.25^a^	5.89 ± 0.09^c^	6.85 ± 0.22^b^	6.35 ± 0.04^bc^	6.63 ± 0.18^b^	<0.001^***^
GSH (U/g)	26.21 ± 0.90^a^	14.72 ± 0.69^c^	21.27 ± 0.85^b^	22.59 ± 0.39^b^	23.33 ± 0.15^b^	<0.001^***^

Data are presented as (Mean ± SE). *n* = 3 chickens; Mean values with different superscript letters in the same raw are significantly different at (*p* ≤ 0.05). *p ≤ 0.05, **p ≤ 0.01, ***p ≤ 0.001.

#### 3.2.2 Assessment of inflammatory parameters

**Figure 3:**
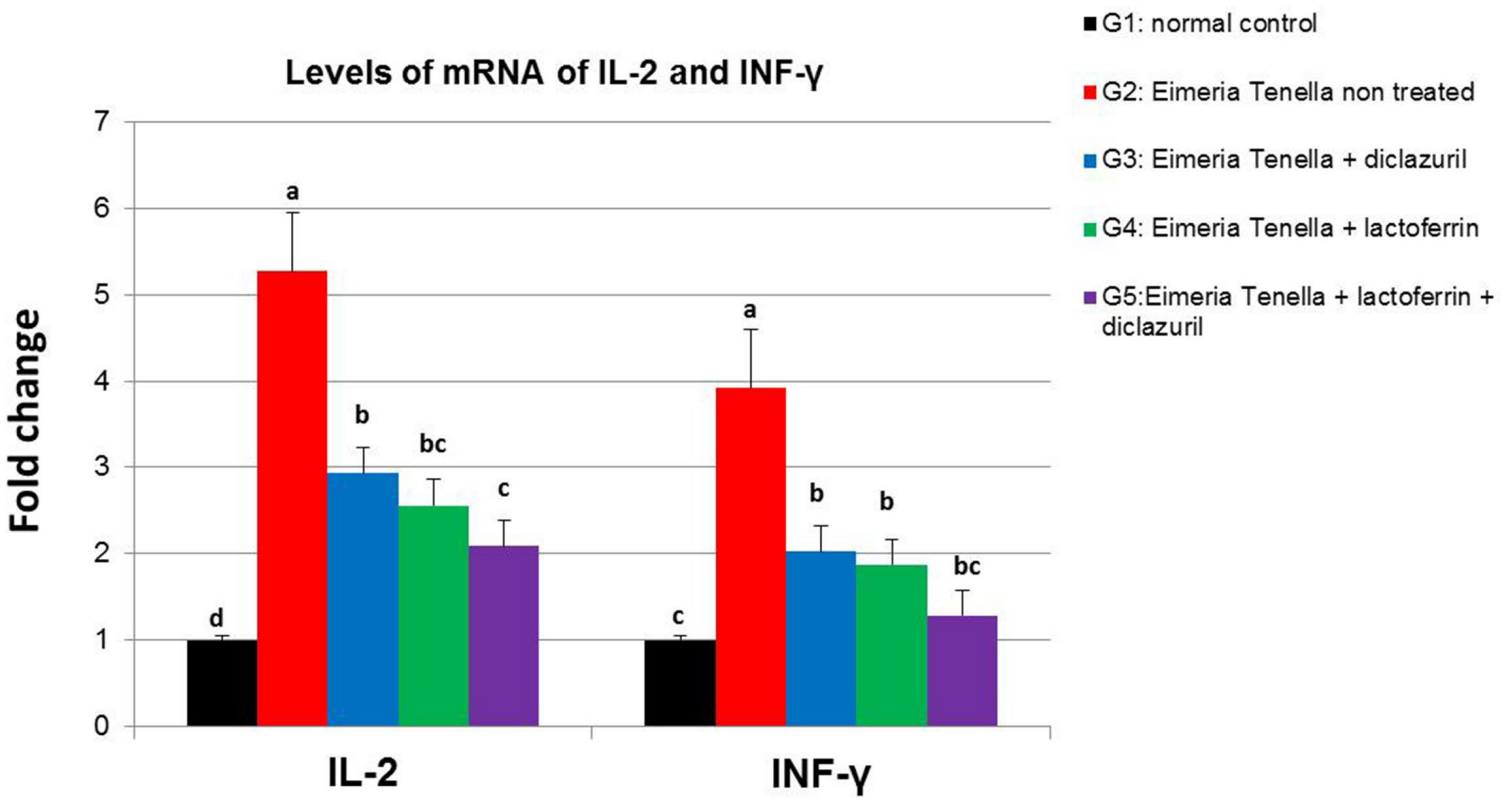
Graphical presentation of real-time quantitative PCR analysis of the expression of IL-2 and INF-γ genes liver (*p* < 0.001^***^) of *Eimeria tenella* induced coccidiosis in chickens following treatment by diclazuril and/or lactoferrin calculated by 2^−ΔΔCt^ method.

Figure 3 illustrates the high (*p* < 0.001) up-regulation of IL-2 and INF-γ liver gene expression in the *E. tenella* non-treated group (G2) compared with the healthy group (G1). Meanwhile, diclazuril and/or LF-treated groups (G3-G5) showed a high (*p* < 0.001) down-regulation of IL-2 and INF-γ compared with the *E. tenella* non-treated group with additive effect of both treatments LF and diclazuril in G5.

## 4 Discussion

Avian coccidiosis remains one of the major parasitic diseases that have a global negative economic impact on the poultry industry (57, 58). The control of coccidiosis mainly relies on the application of prophylactic coccidiostats in the feed (59). However, the development of anticoccidial resistance to the chemical anticoccidial feed additives and potential harmful effects on human health hazards, employ an urgent need to determine the safe alternatives for controlling avian coccidiosis. The current study unveils the potential efficacy of LF and diclazuril in treating *E. tenella* infection in broiler chickens. This is established through the comprehensive assessment of parasitological, biochemical, immunological, and histopathological findings, coupled with evaluations of growth performance, clinical signs, lesion scoring, and mortality rate. To the best of our knowledge, this is the first report to investigate the potential benefits of the use of both materials to treat coccidiosis.

In the present study, the peak of oocysts shedding occurred on the 4th to 5th day post-infection (approximately 108 h post-infection). This finding closely aligns with Ahad et al. (60), who recorded the highest oocyst count on the 4th day post-infection. However, other studies have documented variations in the peak shedding day. In this respect, Choi et al. (61) recorded the peak on the 5th to 6th or 6th to 7th day post-infection, depending on the infection dose. Also, Gong et al. (62) observed the peak on the 6th day post-infection, while El-shazly et al. (33) noted it on the 9th day post-infection. These discrepancies in peak oocyst shedding times are likely due to variations in *E. tenella* inoculation dosages (61), as higher dosages may shorten the asexual and sexual generations of the parasite (63). Consequently, earlier peaks in oocyst shedding have been observed with the highest inoculation dosages (61). It is also important to consider that the pathogenicity of coccidiosis is influenced by a combination of host and parasite factors (64). These factors encompass the inherent pathogenicity of the *Eimeria* species, the reproductive capacity of the infecting species, the age and quantity of ingested oocysts, and the chickens’ susceptibility. Each of these elements, either individually or in combination, can influence the timing of peak oocyst shedding (65–67).

while, there is a significant decrease in the oocyst number in the LF-treated group (G4) occurred because LF is a natural multifunctional protein that inhibits the growth of parasites through iron chelation and the ability of its peptides (Lactoferricins) to pass through the protozoal cell membrane and nuclear envelope, and so treated sporozoites with 1,000 μg/mL bLfcin showed less infectivity and less penetration into host cells than untreated sporozoites, as mentioned by Omata et al. (27) and Reyes-López et al. (68). Interestingly, LF is a harmless protein that could be used in combination with low doses of other drugs. Treatment with both LF and diclazuril in the present study enhanced the anticoccidial efficacy and resulted in a significant reduction of oocyst count compared to G3.

As shown in this present study, the untreated group (G2) exhibited a range of clinical signs of infection on the fifth-day post-infection, such as reduced appetite, lethargy, depressive behavior, weight loss, and the presence of bloody diarrhea, which have been reported in the previous research (69) as common clinical signs in coccidia infection. These signs could be explained by the hypothesis that the environment of the digestive tract of the host stimulates oocyst excystation in the gizzards, resulting in the release of sporozoites that invade and kill cells of the intestinal mucosa and begin the reproductive cycle of the parasite. Meanwhile, the infected groups treated with diclazuril and/or LF (G3-G5) showed milder degrees of symptoms, which might be attributed to the anticoccidial activity of diclazuril (70). In the present study, G2 experienced an obvious decrease in BW and BWG which could be attributed to less feed consumption and breakdown of the intestinal integrity as an absorptive membrane and would result in much less effective nutrient digestion and feed utilization (71). These results agreed with Choi et al. (61) who attributed these findings to the deleterious effects of the *Eimeria* parasite that invades the intestine of the chicken and retards its intestinal function. In contrast, all treated groups with diclazuril and/or LF (G3-G5) showed a significant increase in BW and BWG. Similar results were reported by El-Azm et al. (72) and Iraee et al. (73) who demonstrated increased BW and BWG after treatment of diclazuril in drinking water for 3 successive days in chickens infected with *Eimeria* species. The possible explanation is elevated activities of corresponding enzymes, amylase, and lipase, besides the anticoccidial activity of diclazuril, which increases villi height, compensating for the poor feed efficiency caused by *Eimeria* spp. infection (74). As shown in the present study, feeding on LF beginning from the first day of age significantly increased BW and BWG compared to those observed in the control groups (G1 and G2). These results were confirmed by a previous study (75), recording an improvement in BW and BWG by LF supplementation, which may be related to improving nutrient digestibility, enhancing nutrient uptake (76), and triggering mucosal cell proliferation and its differentiation (22). The LF-treated groups (G4, G5) showed that inhibition of the growth of invading pathogens in the gastrointestinal tract may indirectly increase the health status and growth performance of birds during the rearing period (77), reflecting the antimicrobial activity of LF by iron-sequestering activity from pathogens (19).

In the current study, infection by *E. tenella* caused acute damage in chickens’ caecum in infected non-treated chickens (G2) combined with the presence of various developmental stages of coccidia, necrotic enterocytes, and leukocytic infiltrations in the cecal epithelial cells. Similarly, previous research (30) reported the presence of lymphocytic infiltration and macrophage with different stages of the coccidian parasite in cecal post-*E. tenella* infection. In contrast, treatment of infected chickens with diclazuril and/or LF (G3-G5) showed protective effects against *E. tenella* damaging effects by attenuating cecal damage. This present finding is consistent with previous research (78) reporting that diclazuril alleviated the damage of caecum caused by *E. tenella* and killed all the developmental stages of *E. tenella,* besides causing a significant activity in halting coccidial cycle development within the treated chickens (79), together consistent without histopathological findings. Meanwhile, the infected treated groups (G3-G5) experienced restoration of these reported histopathological changes that might be attributed to the anticoccidial effect of diclazuril and LF in all treated groups by improving the antioxidant and immune systems in chickens.

The present study revealed that the measured hematological parameters (PCV%, Hb%, and RBCs count) were dramatically decreased in the control positive group (G2), which may be the result of second-generation schizont damage, leading to substantial mucosal blood vessel damage and blood loss (80). Similarly, a previous study (81) recorded a significant reduction in hematological parameters in *E. tenella*-infected chickens owing to the acute bleeding and tissue rupture in the mucosa induced by an attack of the parasite. All treated groups (G3–G5) showed improvement in hematological parameters, which is supported by a previous work (82) that demonstrated that an LF supplementary diet combined with iron was capable of improving hematological indices. Additionally, diclazuril revealed a noticeable activity in interrupting the cycle of coccidial development within the treated birds, especially when applied on the first day of blood appearance in the feces (79). Meanwhile, in the present study, there was a significant increase in WBCs and heterophils, besides decreased lymphocyte count in the infected non-treated group (G2) compared with the control healthy group (G1). These results could be explained by induction of the expression of inflammatory response to infection (83), which is usually associated with neutrophilia and lymphopenia (84). In stark contrast, infected chickens supplemented with diclazuril and/or LF (G3-G5) showed a significant reduction in total WBCs and heterophils compared with the infected non-treated group (G2), illustrating the immunomodulatory roles of diclazuril and LF. Similarly, Ward and Conneely (85) recorded that LF showed a regulatory role in the immune system *in vivo*.

Total protein and its major components, albumin, and globulins, perform a key role in the activity of the immune system in various species (86). Therefore, blood chemistry was greatly affected by *the Eimeria* challenge in this study. The positive control groups experienced a decrease in serum TP, albumin, alpha 1, and gamma globulins, while there was a significant increase in AST, ALT, ALP, uric acid, and creatinine levels which might be owing to the malabsorption of protein and other nutrients from the intestine because of hemorrhage and mucosal damage in these birds (2). The reduction in feed intake and absorption of proteins elevates protein catabolism in muscular tissues, leading to muscle degradation and eventually elevating serum levels of ALT, AST, creatinine, and uric acid (33). ALP activity may be a sensitive sign of the pathogenesis in caecum coccidial infection (87). The external hemorrhage can also cause hyperactivity of bone marrow to release excess blood corpuscles, which increases serum ALP level (88). This could also be related to cell destruction owing to the parasitic infection, that triggers the escape of enzymes into the bloodstream (89). The destruction of cells induces the release of many enzymes like AST, ALT, and ALP into serum, and therefore, their levels are elevated depending upon the kind and extent of destruction (90). In contrast, those groups treated with diclazuril and/or LF (G3-G5) showed improvement in all these parameters when compared with the infected non-treated group (G2). These results are consistent with previous work (91) that reported improvement of the hepato-renal functions via a significant reduction in loss of protein, tissue damage, and enzyme release by LF, which is expressed by elevation of serum TP, albumin, and globulin and decrease in ALP, ALT, AST, and creatinine levels. Serum total protein expressed by electrophoresis involves albumin and alpha, beta, and gamma globulins. The globulins are involved in several diagnostically important acute phase proteins. In the present study, chickens treated with LF recorded elevated gamma globulins, reflecting its immunomodulatory and preventive effects in the liver (92).

The present study reported a highly significant decrease in serum glucose levels in G2 as compared to the normal control group (G1). Similarly, Abd El-Maksoud et al. (93) mentioned that serum glucose was decreased in coccidia-infected chickens, which might be because of anorexia and/or intestinal tract inflammation, preventing absorption of glucose and liver glycogenolysis (94). In addition, the reduction of serum glucose levels may be related to the prevention activities of lipase and amylase in the infected non-treated groups (95). In contrast, diclazuril and LF-infected treated groups (G3-G5) exhibited an increase in serum glucose, lipase, and amylase levels. Similarly, Ghasemi-Sadabadi et al. (96) observed that diclazuril increased serum glucose in broiler chickens, which might be attributed to the roles of LF in reinforcement and increase of intestinal epithelial size and function.

Our results revealed that G2 showed a significant increase in MDA and NO concentration when compared with the normal control group (G1). These results are in accordance with previous investigations (97) reporting an increase in MDA concentration and the marked reduction of the SOD activity in infected birds, indicating the occurrence of oxidative stress owing to infection and the impairment of antioxidant/pro-oxidant equilibrium in favor of pro-oxidants. Oxidative stress related to *E. tenella* in broiler chickens results in a reduction of the levels of GSH, and SOD enzymes which are important for inhibiting the destruction of free radicals throughout coccidia infection (98). Meanwhile, NO levels elevated in G2 in response to pathogenic sporozoite phases that penetrated and inflamed cecal cells (99). On the contrary, treatment of all infected animals with LF and/or diclazuril (G3-G5) showed improvement of all these parameters as markedly reduced MDA and elevated antioxidant enzymes such as CAT and SOD, reflecting the antioxidative effects of LF related to its iron-binding properties by sequestering iron (100).

A high up-regulation in liver gene expression of IL-2 and INF-γ was observed in the positive control group (G2) when compared with the normal healthy group (G1). T cells play a key role in the regulation of cell-mediated immunity by secreting cytokines such as INF-γ, and IL2 and mediates the T immune response in avian coccidiosis. Among the cytokines involved in coccidiosis, IFN-γ is a representative immunomodulator and has received the most attention because of its direct influence on the intracellular development of *Eimeria*. Similar results were observed in *E. tenella*-infected chickens (G2) in our present study. Upregulated expression of IFN-γ was recorded in cecal tonsils, spleens, and post-primary as well as secondary *E. tenella* infections. IFN-γ was found to be released by mitogen or antigen-enhanced specific T cells that are present in the blood of chickens infected with *Eimeria* (101). In contrast, the other treated groups (G3-G5) might have experienced an improvement in the immune condition of chickens. Similarly, a previous study (102) recorded that the immune response modulates using LF feed additives as immunostimulant prebiotics. This improvement of liver immune gene expression in chickens fed LF might be related to enhancing birds’ immunity against infectious diseases. However, information on the influence of LF additives on immune response and its impact during infection disease in poultry is limited.

Our results demonstrated that Group 5 (G5) showed significant improvement in oxidant/antioxidant status, enhanced cecal tissue health, and reduced oocyst shedding. These findings suggest that the prebiotic enhanced the anticoccidial effect of diclazuril, consistent with the results reported by Farag et al. (103). Similarly, Barberis et al. (104) highlighted the efficacy of combining prebiotics and anticoccidial drugs in controlling coccidia infections in chickens, noting improvements in immune responses and growth performance. Given the rising drug resistance in *Eimeria* strains, the combination of prebiotic LF with anticoccidial drugs presents a promising strategy to enhance drug efficiency and control coccidia infections, potentially serving as an effective alternative in managing these infections. However, it is important to consider that the present study has some limitations, such as feed intake and feed conversion rate were not measured, which could have further strengthened the findings. Additionally, while the study focused on measuring antioxidant parameters in liver tissues, similar to numerous previous studies, it would have been beneficial to also assess these parameters in cecal tissues for a more comprehensive analysis.

## 5 Conclusion

The current study concluded that LF might possess a potent anticoccidial and anti-inflammatory activity as it reduced the lesion severity and the oocysts shedding. However, this should be preceded by future expanding its evaluation in field. Interestingly, the combined use of diclazuril with LF resulted in superior improvement of oxidant/antioxidant status and of caecal tissues, as well as lower lesion scores and oocyst shedding, reflected by histopathological findings of caecal tissues. Clearly, LF can be recommended to be used as a potential natural anticoccidial in broilers for combating the reported side effects of common anticoccidial agents. Also, it could be concluded that LF can be used as a natural alternative growth promoter in broiler chickens as it has appositive effects on growth performance. Further research is recommended to explore the main mechanistic pathways involved into the anticoccidial actions of LF and the potential application in similar diseases of veterinary and medical importance.

## Data availability statement

The original contributions presented in the study are included in the article/supplementary material, further inquiries can be directed to the corresponding author.

## Ethics statement

The animal study was approved by AHRI in conformity with the ARC and IACUC committee (ARC, AHRI, IACUC, 85/23). The study was conducted in accordance with the local legislation and institutional requirements.

## Author contributions

AGA: Conceptualization, Data curation, Formal analysis, Investigation, Methodology, Project administration, Resources, Software, Supervision, Validation, Visualization, Writing – original draft, Writing – review & editing. NE: Conceptualization, Data curation, Formal analysis, Investigation, Methodology, Project administration, Resources, Software, Supervision, Validation, Visualization, Writing – original draft, Writing – review & editing. MH: Conceptualization, Data curation, Formal analysis, Investigation, Methodology, Project administration, Resources, Software, Supervision, Validation, Visualization, Writing – original draft, Writing – review & editing. SS: Conceptualization, Data curation, Formal analysis, Investigation, Methodology, Project administration, Resources, Software, Supervision, Validation, Visualization, Writing – original draft, Writing – review & editing. AG: Conceptualization, Data curation, Formal analysis, Investigation, Methodology, Project administration, Resources, Software, Supervision, Validation, Visualization, Writing – original draft, Writing – review & editing. MM: Conceptualization, Data curation, Formal analysis, Investigation, Methodology, Project administration, Resources, Software, Supervision, Validation, Visualization, Writing – original draft, Writing – review & editing. AAMA: Data curation, Formal analysis, Funding acquisition, Software, Validation, Writing – review & editing. HA: Data curation, Formal analysis, Funding acquisition, Investigation, Resources, Software, Validation, Writing – review & editing. EE: Conceptualization, Data curation, Formal analysis, Funding acquisition, Investigation, Methodology, Resources, Software, Validation, Visualization, Writing – original draft, Writing – review & editing.
